# Improved Nutritional Knowledge in the Obese Adult Population Modifies Eating Habits and Serum and Anthropometric Markers

**DOI:** 10.3390/nu12113355

**Published:** 2020-10-30

**Authors:** Lourdes López-Hernández, Francisco Miguel Martínez-Arnau, Pilar Pérez-Ros, Eraci Drehmer, Ana Pablos

**Affiliations:** 1 Department of Nursing, Universidad Católica de Valencia San Vicente Mártir, 46007 Valencia, Spain; lourdes.lopez@ucv.es (L.L.-H.); francisco.m.martinez@uv.es (F.M.M.-A.); 2 Hospital Universitario y Politécnico La Fe, 46026 Valencia, Spain; 3 Department of Physiotherapy, Universitat de València, 46010 Valencia, Spain; 4 Frailty and Cognitive Impairment Research Group (FROG), Universitat de València, 46010 Valencia, Spain; 5 Department of Nursing, Faculty of Nursing and Podiatry, Universitat de València, 46010 Valencia, Spain; 6 Department of Basic Sciences, Universidad Católica de Valencia San Vicente Mártir, 46007 Valencia, Spain; eraci.drehmer@ucv.es; 7 Department of Physical Activity and Sport Sciences, Universidad Católica de Valencia San Vicente Mártir, 46007 Valencia, Spain; ana.pablos@ucv.es

**Keywords:** obesity, diet, energy expenditure, macronutrients, micronutrients, health knowledge, attitudes

## Abstract

Multicomponent lifestyle interventions achieve good results in the management of obesity among the adult population. However, their implementation in certain populations poses difficulties. A good level of nutritional knowledge enables people to make changes in their diet that improve their health. This study aims to assess the relationship between nutritional knowledge and nutritional parameters such as dietary intake, anthropometric parameters and biomarkers. A before–after, non-randomized interventional study involving a two-monthly nutritional educational intervention was carried out over 8 months. Anthropometric and biomarker data were collected, and nutritional knowledge was evaluated using the Bach questionnaire and food frequency questionnaire (FFQ). The study comprised 66 overweight and obese adults with mean age of 50.23 years. Females predominated (84.8%). At the end of the intervention, nutritional knowledge increased significantly, with a significant reduction in the consumption of sweets, soft drinks, high-fat products, and processed meats, and an increase in the intake of lean meat and poultry. A 3% decrease in body weight was observed. An intervention for the management of obesity in the adult population based on nutritional education achieves weight loss, modifications in eating habits and reduction of fat intake. Increased nutritional knowledge is associated with healthier eating habits and a decreased cardiovascular risk.

## 1. Introduction

The prevalence of overweight and obesity has increased in recent years to nearly one-third of the world population [1]. No country has managed to stop this trend [2]. In Spain, in 2016, the prevalence of overweight in the adult population was 39.5%, with a prevalence of non-morbid obesity of 19.5% [3]. Overweight and obesity can cause premature disability and death, and is related to an increased risk of cardiometabolic diseases, type 2 diabetes, dyslipidemia, osteoarthritis, dementia, depression and certain types of cancer [4,5]. Excess body fat is a complex and multifactorial problem of genetic, behavioral, socioeconomic and environmental origin [1,6], i.e., both modifiable and non-modifiable factors are involved in development of the disease. The definition of obesity and overweight depends upon the method used to determine the presence of obesity [7]. In 2017, the European Association for the Study of Obesity proposed new diagnostic criteria for obesity based on three dimensions: etiology, degree of adiposity and health risk. Body mass index (BMI) was proposed as an etiological marker, because obesity in most cases is of multifactorial origin. Considering the difficulties inherent in assessing risk on the basis of BMI alone, the risk to the health of an obese patient requires further specification, and physicians need to be provided with tools (e.g., algorithms, software) in order to reliably and validly assess health risk [8,9]. The combined use of anthropometric parameters such as BMI and waist circumference (WC) would make it possible to identify people at greatest cardiovascular risk. However, the most appropriate approach undoubtedly is to perform a comprehensive nutritional study [10]. This includes dietary, clinical and anthropometric assessments; screening instruments; and the analysis of biochemical markers—all of which require considerable investment in terms of time and resources, as well as interdisciplinary work [11,12].

Obesity is known to be associated with health-related behaviors [7]. It is a multifactorial disorder caused by both non-modifiable factors such as age, sex, socioeconomic status, ethnic group and familial characteristics, and modifiable factors such as level of physical activity, dietary patterns and nutrition knowledge [13]. Treatment therefore should be aimed at reversing the modifiable factors [14]. Diet is a major modifiable risk factor for cardiometabolic conditions such as dyslipidemia and type 2 diabetes [5,15]. Nutritional knowledge, attitudes and eating self-regulation are important determinants of overweight and obesity [16]. Eating behavior is one of the modifiable factors leading to overweight and obesity. Knowledge about what should be eaten and the importance of healthy food habits is necessary in order to change and improve eating behavior [13]. One of the diets with the strongest evidence for weight loss in obese people is the Mediterranean diet, which also reduces cardiovascular risk and mortality. The Mediterranean diet is characterized by a high intake of fruits and vegetables, cereals and monounsaturated fats (primarily from olive oil), a moderate consumption of poultry, fish and dairy products, and little to no consumption of red meat [17,18].

Based on the evidence found in the literature on the management of obesity, the existing clinical guides underscore several important issues: (1) Recognition of obesity as a chronic disease by healthcare providers; (2) Assessment of individuals living with obesity, using appropriate measurements; (3) Discussion of the core treatment options; (4) Agreement with individuals living with obesity regarding the goals of therapy; and (5) Implication of healthcare providers in the continued follow-up and reassessment of obese individuals [10].

Several studies indicate that the gold standard for obesity management and prevention consists of multicomponent lifestyle intervention programs (nutrition, physical activity and cognitive-behavioral intervention) [19,20,21,22]. However, at the same time there are barriers to adherence to programs of this kind [23], since not all the population can afford this type of intervention, due to the time that must be spent exercising, or because of a lack of knowledge of dietary management [24,25], the many erroneous data found on the internet, with false myths and diets, etc. [26,27]. A systematic review emphasized the need to focus on long-term interventions with a duration of ≥5 months and involving ≤3 focused objectives, randomization, the use of theories and fidelity as factors that enhance success [28].

The difficulty of adherence to multicomponent programs, the need for long-term intervention studies, and previous studies in different age ranges point to the need to develop programs to prevent obesity and improve nutritional education [29]. Most obesity prevention programs achieve favorable outcomes, with multicomponent programs being more effective in this regard. However, positive results have also been reported with single interventions such as physical exercise or nutritional education [30,31].

Considering the barriers facing the implementation of multicomponent programs, the present study was carried out to determine the effect of a nutritional education program over 8 months in obese adults on eating habits, anthropometric parameters and serum biomarkers.

## 2. Materials and Methods

### 2.1. Design

This was a before–after non-randomized interventional study. The inclusion criteria were: age 20–70 years, BMI > 25 kg/m^2^, and lack of regular physical exercise. The exclusion criteria were: blindness and deafness, and cognitive impairment diagnosed by a physician. The study was carried out between October 2014 and May 2015.

The G*Power 3.1 application (Universität Kiel, Kiel, Germany) was used to calculate the required sample size. To detect a 3 kg difference after the intervention with a 5% alpha error (α = 0.05) and a statistical power of 80% and a standard deviation of 5.8 [32], a sample size of 45 participants was needed. Assuming a 10% attrition rate, the final sample size was established as 50 individuals.

The subjects were recruited in the months prior to the start of the school year in Spain (June–September) in 2014. In order to encourage participation, various advertising media were used to disseminate the information. As a result, 80 participants were recruited at this stage.

The Human Research Ethics Committee of the University of Valencia (Valencia, Spain) approved the project (procedure number: H1427122754390). All participants were informed before and after recruitment about the intervention and personal data protection, and written informed consent was obtained in all cases.

### 2.2. Data Collection

The assessments were carried out at the Universidad Católica de Valencia San Vicente Mártir (Valencia, Spain) in the mornings between 8 am and 11 am over 5 days (Monday to Friday). The professionals in charge of the measurements were two nurses in the case of peripheral blood samples, and the anthropometric measurements were performed by the same evaluator with ISAK level II accreditation, in order to avoid variability. A maximum of three participants were scheduled each hour. In order to guarantee the privacy of the patients during assessment, a circuit of separation stops was established, and the same order was always followed in the collection of the data.

Sociodemographic data were collected: sex, age, marital status and educational level. This was followed by the nutritional evaluation, comprising anthropometric parameters and blood markers. In addition, data were collected using a food frequency questionnaire (FFQ), with calculation of the dietary macronutrients and micronutrients using the Easy Diet^®^ tool (Academia Española de Nutrición y Dietética, Pamplona, Spain). Physical activity was categorized as mild or moderate according to the World Health Organization (WHO) (Geneva, Switzerland) [33].

### 2.3. Nutritional Assessment

The nutritional assessment consisted of anthropometric data and blood biomarkers. For the anthropometric evaluation, we recorded body weight, height and BMI, and the percentage of body fat was obtained from the folds at triceps, biceps, subscapular, iliac crest, suprailiac and abdominal level [34]. In addition, the waist-hip index and waist-height ratio were calculated after measuring the waist and hip circumferences. The values of the biomarkers were determined from samples of peripheral blood obtained after a fasting period of at least 8 h. The biomarkers analyzed were glucose, total cholesterol, HDL-cholesterol, LDL-cholesterol, triglycerides and C-reactive protein (CRP).

### 2.4. Nutritional Knowledge

The instrument used to assess participant nutritional knowledge was the Nutrition Knowledge Questionnaire [35]. The latter consists of 53 items addressing nutritional content related to food properties, composition and function, dietary recommendations, and consequences of obesity. The subjects were required to answer “true” or “false” to each question. The resulting score ranged from 0 to 53.

### 2.5. Eating Habits

#### 2.5.1. Food Frequency

A validated Food Frequency Questionnaire (FFQ) [36] was used to estimate dietary intake. This instrument was previously used to conduct nutritional studies in the Spanish population. It is a self-administered questionnaire, and the subjects are required to answer 93 items distributed into nine groups of foods, with a standard unit or serving. For each item the subjects must select one of the nine possibilities related to frequency of consumption, ranging from “never or less than once a month” to “six or more times a day”.

#### 2.5.2. Easy Diet^®^

To estimate the consumption of macronutrients and micronutrients, standard servings obtained from the FFQ were converted using the registered Easy Diet^®^ program (Academia Española de Nutrición y Dietética, Pamplona, Spain). This program allows us to enter the amount of food eaten and performs a nutritional analysis to yield the macronutrients and micronutrients consumed.

### 2.6. Intervention

Based on the evidence on the management of obesity in adults, the following recommendations were followed [10]: (1) Recognition of obesity as a chronic disease by healthcare providers–the intervention program being carried out by a nurse; (2) Assessment of individuals living with obesity, using appropriate measurements and performing a complete nutritional assessment, allowing data to be obtained in a comprehensive way; (3) Discussion of the core treatment options: it was explained that the program would consist of a nutritional education intervention; (4) Agreement with individuals living with obesity regarding the goals of therapy: all participants agreed to participate in this type of intervention; and (5) Implication of healthcare providers in the continued follow-up and reassessment of obese individuals, with intervention and follow-up being carried out over 8 months.

The intervention consisted of 15 fortnightly nutritional education workshops conducted by a nurse and nutritionist with more than 10 years of teaching and research experience in the field of nutrition. The sessions were held in the classrooms of the Inmaculada Concepción campus of the Universidad Católica de Valencia. The maximum capacity of each workshop was 40 people. The sessions were organized with a theoretical-practical character and lasted 60 min each. In the workshops, aspects of general interest related to nutrition were worked upon, and it was underscored that the participants could apply them in their daily lives. The teaching methodology consisted of about 10 min reviewing the previous session and clarifying possible doubts, followed by about 20–25 min for the content of the session. Afterwards, approximately 20 min were dedicated to a practical session (analysis of the diet, data found on the Internet or study of cases with an easy solution and finally, about 10 min for clarifying possible doubts).

After the assessments were made, the participants were divided into two groups in order to encourage their participation in the workshops and direct contact. The criterion used for group assignment was the calendar preference of the participants. They previously received a calendar of sessions, and each session was held at two different times (in the morning and afternoon) in the same week, to encourage attendance. In these workshops, we worked upon aspects of general interest and easy application in daily life. The complexity and applicability of the contents were established in ascending order. Workshop number 4 (adaptation tips for holidays and extraordinary events) was held just before the Christmas break period, in order to provide useful tips and strategies for holidays such as Christmas, Easter and local celebrations (Supplementary Materials Supplementary file 1. List of sessions).

The nutritional education intervention was supported by psychological reinforcement sessions in which the participants, in coordination with the acquisition of nutritional knowledge, were able to reinforce motivation, goal setting and control of food-related emotions [37].

A series of measures were established to safeguard the fidelity of the interventions. Participants could submit complaints or suggestions. Understanding of the content of each workshop was ensured initially through the study exclusion criteria (i.e., individuals with cognitive impairment were not eligible) and subsequently through practical exercises in the second part of the session. In addition, the participants were provided with a telephone number to call if they had any doubts between the days of the sessions. A minimum session attendance criterion of 80% was established to include the participant in the final results of the study. For this purpose, attendance control was applied in each session. In order to facilitate access to all the information provided in the workshops, participants were given a notebook with a summary including the contents worked upon in the sessions (Supplementary Materials Supplementary file 2. Summary of contents).

In addition, two follow-up sessions on body weight and waist and hip circumference were held in the first week of month 3 and in the first week of month 6 to monitor the variation of these parameters over time.

### 2.7. Data Analysis

The variables were reported as proportions and/or means and standard deviation (SD). Parametric tests (Student *t*-test) were used for the comparison of means, while non-parametric tests (chi-square test) were used for the comparison of proportions. Post-intervention variables were analyzed using the Student t-test with the corresponding 95% confidence interval (95%CI).

The degree of association between nutritional knowledge and the different factors was studied, first by analyzing the correlation between the pre-post change in measurements, and then a linear regression model was constructed to assess the importance of the factors in relation to the change in nutritional knowledge after the intervention program.

We first considered the complete model with all the variables found in the bivariate analysis to be significantly associated to change in nutritional knowledge. In a second step, we eliminated from the model all those variables that failed to produce an important change (defined as the absence of an adjusted effect of >10%), or which did not result in an improved standard error of the estimate on adjusting the model without such variables. Consensus was sought among the investigators in cases where two or more subsets of variables with the same degree of fit were obtained.

Based on these criteria, the variables included in the model were: age, sex, physical activity level, and change referred to the consumption of nuts, highly saturated fats, fats, sweets, fish, eggs, legumes, dairy products, olive oil, vegetables, fruits, cereals, water, soft drinks, wine and beer, as well as change referred to glucose, cholesterol, LDL-cholesterol, HDL-cholesterol, triglycerides, lean meat, and CRP. The dependent variable was the change in nutritional knowledge.

The study data were entered in MS Excel spreadsheets, and data analysis was performed using the SPSS^®^ version 23.0 statistical package (IBM SPSS Statistics) (SPSS Inc., Chicago, IL, USA).

## 3. Results

Of the total 97 participants initially evaluated for inclusion in the study, 31 (31.9%) were excluded due to the following reasons: 6 (19.4%) failed to meet the inclusion criteria, 12 (38.7%) declined to participate, and 13 (41.9%) failed to come to the assessments. The final study sample thus comprised 66 participants (69.1%). Following the intervention, four subjects were excluded from the analysis because they had not attended 80% of the sessions.

The mean age of the participants was 50.23 ± 11.9 years, and females predominated (*n* = 56, 84.8%). A total of 18.2% (*n* = 12) lived with their partner. The illiteracy rate was low (9.1%, *n* = 6). A total of 60.6% of the participants (*n* = 40) reported moderate weekly physical activity, with aerobic and anaerobic exercise lasting between 150–300 min on 2–3 days a week (Table 1).

Cronbach’s alpha of the Nutrition Knowledge Questionnaire was α = 0.726 (rated as acceptable). A mean score of 20.47 ± 9 was recorded at baseline. At the end of the nutritional education program, a significant increase in level of knowledge was observed (mean difference [MD] = 10.81; 95%CI: 7.76–13.87; *p* < 0.001).

Statistically significant changes were observed for all the anthropometric parameters, with a weight loss of 3% and a significant decrease in BMI, despite the persistence of values indicating obesity, with an average of over 30 kg/m^2^. Similarly, waist circumference, waist-hip index, waist-height ratio and percentage body fat were reduced, though the scores likewise continued to indicate obesity (Table 2). In contrast, although the biomarkers were seen to decrease, statistical significance was only reached for HDL-cholesterol (Table 2).

Similarly, changes were observed in food frequency after the intervention, with a statistically significant increase in the consumption of lean meats and a decrease in the consumption of nuts, olive oil, bread, cereals, sweets, soft drinks, fatty meats and cold meats (Table 3).

The consumption of macro- and micronutrients was also analyzed. Lower energy consumption was observed, and consequently a decrease was recorded in intake of the three macronutrients, though the amount of carbohydrates and fats in all subtypes proved significant (Table 4).

In contrast, no major differences were observed in the consumption of micronutrients, except for sodium, which decreased by an average of 1455 mg per day (Table 4).

**Figure 1:**
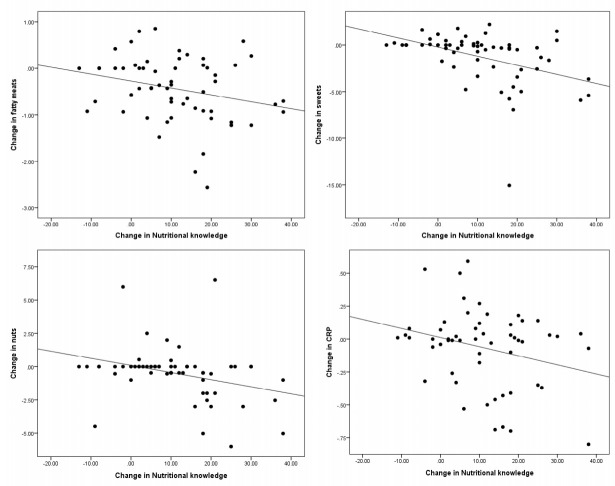
Correlation between change in nutritional knowledge and variation in food servings and C-reactive protein (CRP).

In order to define the relationship between the increase in nutritional knowledge and improvement of the anthropometric parameters, biomarkers and food frequency, correlations were established between the gain in knowledge and the differences in means of the rest of the parameters (Figure 1).

A correlation was found between the increase in nutritional knowledge and the decrease in consumption of fatty meats and cold meats (r = −0.265; *p* = 0.041), sweets (r = −0.422; *p* = 0.001) and nuts (r = −0.317; *p* = 0.014). The furthest points corresponded to subjects with a weight loss greater than the group mean in the fatty meats and sweets. In addition, a correlation was found with the decrease in CRP levels (r = −0.278; *p* = 0.038) (Figure 1). No correlations were observed between the other parameters.

Lastly, a linear regression analysis was made to quantify the relationship between the increase in nutritional knowledge and the differences between the observed values (Table 5).

The increase in nutritional knowledge was related to a decrease in CRP and the consumption of dairy products, nuts, sweets and fatty meats and cold meats, and to an increase in the consumption of fruits, vegetables and water. The regression coefficient proved statistically significant (F = 6.296; *p* < 0.001), with R = 0.797 and R^2^ = 0.635.

## 4. Discussion

In the present study, we evaluated the effect of a nutritional education program upon different anthropometric, serum and food consumption parameters over a period of 8 months in an obese adult population. After completion of the program, an increase in level of knowledge and improvement of the nutritional and serum parameters were recorded, with a modification of food intake. There was a change in several eating habits, since the type of meat consumed was significantly modified, with a decrease in fat in favor of lean meat, in addition to a decrease in sweets, cereals, and soft drinks. According to the WHO [39], the main objectives of a healthy diet include increased healthy fat intake, a high intake of fruit, an increase in the consumption of protein of vegetable origin (legumes and nuts), and the minimization of alcohol intake. The present study found a decrease in daily caloric intake, with a slight increase in protein and carbohydrates and a decrease in fat—with saturated fats showing the highest percentage decrease. Despite the observation of an increase in the consumption of vegetables, legumes, lean meat and fish proteins instead of fatty meats, we also recorded a decrease in the servings of foods considered to be healthy, such as nuts and olive oil. In addition, the increase in alcohol consumption was associated with an increase in the consumption of red wine to the detriment of distilled beverages and beer. Our results did not reflect a complete change in dietary habits towards a healthy diet. The literature indicates that an increase in knowledge is not always correlated to changes in eating habits, due to the existence of fragmented knowledge delivery, i.e., the lack of a relationship among the characteristics of an adequate diet, the concept of obesity and its cardiovascular consequences. In fact, the large body of information currently available on the Internet, television and social networks does not favor the applicability of knowledge in changing eating habits [40].

Despite the observation of a decrease in fruit consumption, the daily intake was higher than the recommended level of intake. A slight increase in vegetable proteins was also found in the form of legumes. The reduction in the percentage of fat in daily intake largely corresponded to saturated fats, though an undesirable effect was also found in the form of a decrease in the intake of heart-healthy fats (olive oil and nuts), related in turn to a decrease in HDL-cholesterol. Given the results obtained, the intervention carried out should be analyzed in depth, and more time should be dedicated to explaining the difference between the types of fats in future interventions [40].

The decrease in the consumption of fatty meats achieved a decrease in saturated fats and also an increase in lean meats and fish. However, such fish consumption was similar or slightly higher than in other studies in adult and older populations, as opposed to adolescent or childhood populations [41,42], with lower fish intake values. In addition, the fact that Valencia is a coastal city could explain the availability of daily fresh fish in supermarkets, contributing to the increased weekly fish intake. The availability of those foods that form part of the Mediterranean diet in our sample could also have facilitated new eating habits, with both quantitative and qualitative effects [43].

The percentages of daily carbohydrates were slightly higher after the intervention even though the daily servings were lower. This could be due to the decrease in the consumption of refined cereals, maintaining the consumption of grain cereals [44,45], since high fiber intakes were also observed from the beginning of the study.

A decrease was also achieved in sweets and soft drinks. Increased awareness and better distinction between beverages available on the market could have reduced the consumption of sweets and beverages [46,47]. Consumers could control consumption with an appropriate strategy such as using different low-calorie and non-calorie sweeteners as a tool to reduce the sugar and calorie content of foods and beverages [46]. These changes could define the diet as being more healthy from the cardiological perspective [17].

Improvement was observed in the anthropometric parameters. However, despite significant weight loss and the reduction of BMI, the participants remained within obesity ranges. The same was seen to occur with waist circumference and the rest of the anthropometric ratios. The recorded weight reduction of 3% was less than that found in other longitudinal studies, despite the fact that 60% of the sample was engaged in moderate weekly physical activity [48,49,50]. Multicomponent interventions achieve better outcomes than those based on other intervention modalities, though interventions that achieve small reductions in daily calories from fat also afford reductions in weight, BMI and waist circumference [51].

The changes in eating habits were not only evidenced by the analyzed data but also by improvement of the anthropometric and serum parameters. The increase in knowledge was correlated to a decreased consumption of sweets, fatty meats and nuts, and to a decrease in serum CRP. C-reactive protein is a prominent biomarker of insulin resistance and cardiovascular disease, and is regulated by the visceral adipose tissue [52,53]. The serum values corroborate the nutritional intake data. The loss of adipose tissue mediated by dietary changes, physical exercise, liposuction or bariatric surgery is accompanied by decreased adipose tissue and systemic inflammation markers [54]. Although no correlation was observed between weight and fat loss and the different serum markers, this could be due to the fact that the decrease in inflammatory markers is greater with the loss of visceral fat than with the loss of subcutaneous fat [52], and in the present study the analyzed fat loss did not include visceral fat. In this regard, most guides on the management of obesity advise complete nutritional assessments that do not only consider anthropometric data. It is also necessary to collect information that can complement and corroborate the rest of the nutritional data obtained [10,11,12].

The regression analysis carried out on nutritional knowledge correlated food intake and the serum parameters. The relationship between exercise, CRP and nutritional knowledge is crucial for understanding how increased nutritional knowledge acts upon heart-healthy habits. A decrease in the consumption of more harmful foods is related to lower CRP levels and higher levels of nutritional knowledge. This reinforces the idea of the importance of physical exercise and healthy eating for the control of inflammation [55]. Moderate physical activity was also related to an increase in nutritional knowledge. The increase in knowledge and the performance of physical activity are an essential tandem in the management of obesity [13]. Although there is evidence to support this relationship, a recent study indicates that increased nutritional awareness is not related to physical activity [56]. This may be due to the type of physical activity performed and to the type of educational intervention employed.

The present study is not without limitations. The first limitation is the fact that the study design lacks a control group; moreover it was not possible to analyze all the nutritional parameters advised by the clinical guides for conducting a nutritional assessment, due to the costs involved. The FFQ is moreover a self-reporting questionnaire that can generate biased information. The low percentage of men could also bias the results.

## 5. Conclusions

An intervention for the management of obesity in the adult population based on nutritional education achieves weight loss and the modification of eating habits, with a reduction of the intake of saturated fat, sweets and beverages. Lifestyle interventions are the most appropriate strategy for the management of obesity in the adult population, though an educational intervention could be implemented in those populations that have difficulties in adhering to a multicomponent lifestyle intervention.

## Figures and Tables

**Table 1:** Sociodemographic characteristics of the participants at the start of the study.

Variable	Mean (SD)/*n* (%)
Age, years	50.23 (11.9)
Sex, %	
Female	56 (84.8)
Male	10 (15.2)
Marital status, *N*, %	
Partner	12 (18.2)
No partner	54 (81.8)
Educational level, *N*, %	
Illiterate	6 (9.1)
Primary	39 (59)
Higher	21 (31.8)
Physical activity	
Sedentary	26 (39.4)
Moderate	40 (60.6)

Primary: Basic general education. Higher: university education.

**Table 2:** Before-after intervention distribution of the anthropometric parameters and biomarkers.

	Pre Mean (SD)	Post Mean (SD)	MD	95%CI (Lower)	95%CI (Upper)	*p*-Value
Weight (kg)	87.24 (16.45)	84.51 (16.81)	2.73	1.78	3.68	<0.001
BMI (kg/m^2^)	32.87 (5.60)	31.79 (5.69)	1.08	0.71	1.45	<0.001
Waist-hip index	0.98 (0.08)	0.97 (0.07)	0.01	0.00	0.03	0.009
Waist circumference (cm)	109.02 (12.99)	105.32 (13.54)	3.70	2.21	5.18	<0.001
Waist-height ratio	0.67 (0.08)	0.65 (0.08)	0.02	0.01	0.03	<0.001
Fat (%)	41.22 (8.18)	39.68 (7.54)	1.54	0.23	2.85	0.022
Glucose(mg/dl)	100.14 (25.96)	99.70 (25.91)	0.44	−2.53	3.40	0.769
Total cholesterol (mg/dl)	212.30 (24.50)	207.20 (32.03)	5.10	−1.68	11.87	0.137
HDL-cholesterol (mg/dl)	55.51 (13.21)	53.18 (11.93)	2.33	0.92	3.74	0.002
LDL-cholesterol (mg/dl)	128.39 (22.93)	126.92 (29.40)	1.47	−4.97	7.90	0.650
Triglycerides (mg/dl)	142.93 (65.41)	135.93 (55.06)	7.00	−4.81	18.81	0.240
CRP (mg/dl)	0.59 (0.59)	0.52 (0.51)	0.07	−0.01	0.15	0.084

BMI: Body Mass Index; CRP: C-reactive protein.

**Table 3:** Before-after intervention mean differences in food servings consumed.

		Pre Mean (SD)	Post Mean (SD)	MD	95%CI (lower)	95%CI (upper)	*p*-Value
Fish and seafood	3–4 s/week	6.91(3.55)	7.24 (5.34)	−0.33	−1.56	0.90	0.596
Lean meat and poultry	3–4 s/week	3.20 (1.58)	4.23 (3.26)	−1.03	−1.82	−0.24	0.011
Eggs	3–4 s/week	2.52 (1.20)	2.40 (1.08)	0.13	−0.19	0.44	0.427
Legumes	2–4 s/week	2.11 (1.46)	2.30 (2.33)	−0.19	−0.82	0.44	0.547
Nuts and seeds	3–7 s/week	1.50 (1.78)	1.00 (1.80)	0.51	0.00	1.01	0.048
Dairy products	2–4 s/day	2.85 (1.48)	2.74 (2.14)	0.11	−0.38	0.59	0.669
Olive oil	2–6 s/d	2.37 (1.13)	1.79 (1.25)	0.58	0.25	0.92	0.001
Vegetables	More than 2 s/day	4.67(2.12)	5.11 (3.42)	−0.44	−1.26	0.39	0.296
Fruits	More than 2 s/day	4.18 (4.19)	3.86 (3.38)	0.32	−0.53	1.17	0.456
Cereals	4–6 s/day	2.81 (1.37)	2.21(1.31)	0.61	0.24	0.97	0.001
Water	4–8 s/day	1.70 (1.02)	1.87 (1.44)	−0.17	−0.49	0.15	0.295
Wine and beer	Sparingly	0.21 (0.36)	0.31 (0.58)	−0.10	−0.23	0.02	0.114
Fats	Sparingly	0.52 (0.75)	0.46 (0.75)	0.07	−0.13	0.27	0.494
Sweets	Sparingly	2.98 (3.16)	1.74 (1.69)	1.23	0.55	1.92	0.001
Soft drinks	Sparingly	0.52 (0.80)	0.29 (0.45)	0.23	0.10	0.37	0.001
High-fat and processed meats	Sparingly	1.37 (0.67)	0.93 (0.53)	0.43	0.26	0.61	<0.001

s/week servings a week; s/day servings a day.

**Table 4:** Before-after intervention mean differences in food servings consumed.

Macronutrients	Recommendation	Pre Mean (SD)/% cal/Day	Post Mean (SD)/% cal/Day	MD	95CI% (Lower)	95%CI (Upper)	*P*-Value
Energy, kcal/day	2200–2900	2519.29 (759.67)	2118.66 (681.05)	400.63	220.33	580.93	<0.001
Proteins, g/day	80–110	111.33 (32.21)/19.5	105.80(40.14)/21.2	5.53	−4.14	15.21	0.26
Carbohydrates, g/day	300–400	245.51 (100.11)/39.5	209.27 (80.50)/41	36.25	14.13	58.36	<0.001
Fiber, g/day	25–38	35.68(14.79)	35.73 (18.83)	−0.05	−4.79	4.69	0.98
Total fats, g/day	73–97	112.43(36.03)/41	85.70 (33.67)/37.8	26.72	16.92	36.52	<0.001
Saturated fatty acids, g/day	<22	32.22 (11.97)/12.91	24.23 (11.40)/11.8	7.99	4.85	11.14	<0.001
Monounsaturated, g/day	27–49	53.08 (18.24)/19.96	39.26 (16.58)/18.2	13.83	8.90	18.75	<0.001
Polyunsaturated, g/day	<26	18.38 (7.43)/7.96	14.97 (8.10)/7.8	3.41	1.17	5.64	<0.001
Cholesterol, mg/day	300	315.92 (99.11)	298.93 (145.44)	16.99	−16.78	50.77	0.32
**Micronutrients**	**Recommendation**	**Pre Mean (SD)**	**Post Mean (SD)**	**MD**	**95CI% (Lower)**	**95%CI (Upper)**	***p*-Value**
Calcium, mg	1000–1200	1148.56 (438.85)	1096.52 (544.92)	52.04	−75.57	179.66	0.42
Iron, mg	10–18	18.41 (5.61)	18.52 (8.83)	−0.12	−2.27	2.04	0.91
Iodine, μg	120–140	133.18 (40.22)	134.73 (60.84)	−1.55	−15.78	12.68	0.83
Magnesium, mg	300–350	473.56 (153.47)	460.31 (186.91)	13.25	−33.96	60.46	0.58
Zinc, mg	15	12.50 (3.66)	11.58 (4.42)	0.92	−0.13	1.97	0.09
Selenium, μg	55	64.65	52.69	12.96	2.42	25.03	0.02
Sodium, mg	1000–5000	5340.68 (1763.24)	3885.29 (1556.03)	1455.38	943.53	1967.23	<0.001
Potassium, mg	3500	5120.20 (1819.96)	5189.10 (2370.53)	−68.89	−657.19	519.41	0.82
Phosphorus, mg	700	2001.42 (631.76)	1887.43 (815.46)	114.00	−66.00	294.00	0.21
Vitamin B1, mg (Thiamine)	1–1.2	2.06 (0.67)	1.97 (0.77)	0.09	−0.10	0.28	0.35
Vitamin B2, mg (Riboflavin)	1.3–1.6	2.29 (0.68)	2.30 (1.01)	−0.01	−0.23	0.21	0.94
Vitamin B3, mg (Niacin)	14–17	45.30 (13.64)	42.74 (17.13)	2.56	−1.70	6.81	0.23
Vitamin B6, mg (Pyridoxine)	1.2–1.6	2.80 (0.86)	2.90 (1.31)	−0.09	−0.40	0.22	0.56
Vitamin B9, μg (Folic acid)	400	563.12 (235.81)	612.59 (374.31)	−49.48	−142.76	43.81	0.29
Vitamin B12, μg	2	15.70 (9.61)	13.73 (15.31)	1.97	−2.12	6.06	0.34
Vitamin C, mg	60	38.88 (23.19)	42.36 (27.56)	−3.47	−10.05	3.1	0.30
Retinol, μg	600–700	733.18 (77.44)	579.63 (86.31)	153.55	−87.95	395.04	0.21
Carotenes, μg	800–1000	8388.86 (4288.4)	9505.68 (7325.5)	−1116.82	−3048.24	814.60	0.25
Vitamin A, mg	1000–2000	2087.35 (934.65)	2128.41 (1491.2)	−41.07	−399.31	317.18	0.82
Vitamin D, μg	5	2.40 (1.41)	2.47 (1.65)	−0.06	−0.49	0.36	0.76
Vitamin E, mg^6^	15	17.25 (7.72)	15.71 (9.18)	1.54	−0.83	3.91	0.20
Alcohol, s/day	1–2	2.07 (3.74)	2.97 (5.60)	−0.90	−2.09	0.29	0.13
Water, mL	2700–3700	1981.63 (690.57)	2064.88 (913.19)	−83.24	−301.89	135.40	0.45

s/d servings a day. Recommended range: rates for women and men in the Spanish population [38].

**Table 5:** Liner regression analysis.

	Beta	95%CI (Lower)	95%CI (Upper)	*p*-Value
Moderate physical activity	8.67	3.23	14.10	0.003
Change in vegetables	1.17	0.37	1.98	0.005
Change in water	1.8	−0.08	3.69	0.061
Change in CRP	−4.88	−13.22	3.5	0.245
Change in dairy products	−0.92	−1.79	0.27	0.157
Change in nuts	−1.5	−3.22.66	−0.35	0.12
Change in sweets	−1.56	−2.49	−0.64	0.001
Change in high-fat and processed meats	−0.88	−1.68	−0.08	0.03

## Supplementary Materials

The Supplementary Materials are available online at https://www.mdpi.com/2072-6643/12/11/3355/s1. Supplementary file 1: List of sessionsTitle; Supplementary file 2: Summary of contents.
