# Supplementary material for: Improved Nutritional Knowledge in the Obese Adult Population Modifies Eating Habits and Serum and Anthropometric Markers
Source: Nutrients. 2020 Oct 30;12(11):3355. doi: 10.3390/nu12113355 (PMC7693073; doi:10.3390/nu12113355)
Supplement: Supplementary file 1 [file nutrients-12-03355-s001.zip › nutrients-975371-supplementary/Supp File 2. NUTRIENTS_975371_R1.pdf]

NUTRITIONAL KNOWLEDGE WORKSHOP NOTEBOOK

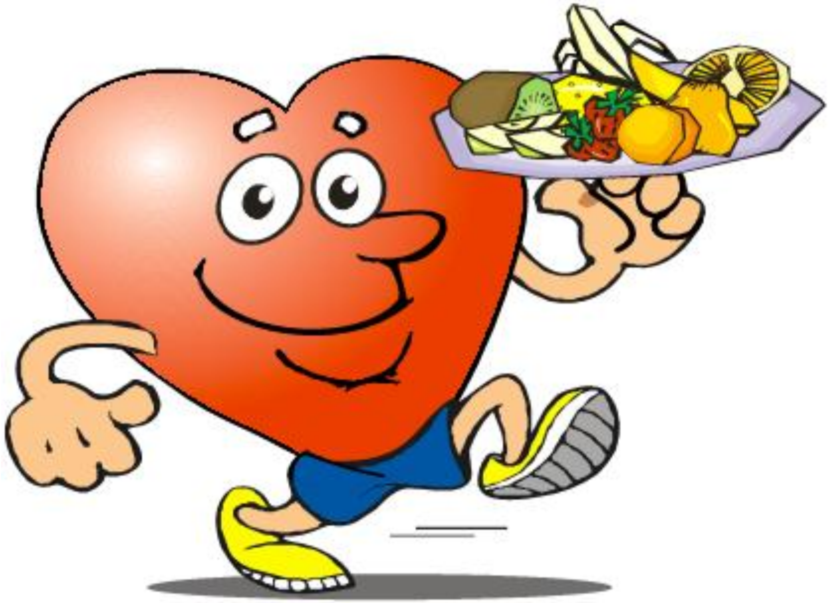

SESSION 2. GENERAL RECOMMENDATIONS FOR AN ADEQUATE FOOD PLAN 2
SESSION 3. WAYS OF COOKING 3
SESSION 4. TIPS FOR ADAPTING FOOD FOR LEISURE AND EXTRAORDINARY EVENTS 1
SESSION 5. RATIONS AND EQUIVALENCES 2
SESSION 6. FALSE BELIEFS AND MYTHS OF FOOD 3
SESSION 8. MACRONUTRIENTS AND MICRONUTRIENTS 11
SESSION 9. OBESITY AND OVERWEIGHT 13
SESSION 10. MIRACLE DIETS 13
SESSION 11. HEALTHY FATS 14
SESSION 13. GUIDELINES FOR MAKING A HEALTHY PURCHASE 1

SESSION 2. GENERAL RECOMMENDATIONS FOR AN ADEQUATE FOOD PLAN

DRINK 1.5-2 L OF WATER/DAY
- GOOD HYDRATION IS FUNDAMENTAL FOR THE BODY
- DRINK WATER EVEN IF YOU DON'T FEEL THIRSTY

USE SOFT COOKING METHODS: GRIDDLE, GRILL, OVEN, STEAM, BOILING, STEW, MICROWAVE
- AVOID COOKING METHODS RICH IN OILS: FRYING, BATTERING, ETC.
- PREFERABLY OLIVE OIL BOTH RAW AND FOR COOKING

MODERATE THE CONSUMPTION OF SALT:
- REPLACE WITH CONDIMENTS, AROMATIC HERBS, SPICES...
- DO NOT ADD SALT TO THE FOOD AFTER COOKING.

REPLACE SUGAR WITH SACCHARINE OR OTHER SWEETENERS.

- SACCHARIN, ASPARTAME, CYCLAMATE, CINNAMON...

IN CASE OF ANXIETY, MAKE USE OF TEAS, FRUIT AND VEGETABLES OR YOGURT.

CONTROL THE DAILY USE OF OIL AND PREFERABLY USE OLIVE OIL (MEDITERRANEAN DIET)

TO MAKE 5 MEALS A DAY.
- DO NOT LEAVE MORE THAN 3-4h between meals to avoid hunger and anxiety.
- SKIPPING MEALS DOES NOT PROVIDE ANY BENEFIT.

FOODS NOT RECOMMENDED: SWEETS, PASTRIES, SNACKS AND SWEET AND ALCOHOLIC DRINKS

PERFORM REGULAR PHYSICAL ACTIVITY

SESSION 3. WAYS OF COOKING

GRILL:

- SIMPLEST METHOD, SPEED AND LOW OIL USE

- USED: GRIDDLE PAN OR GRILL.

- SPICES ARE A GOOD COMPLEMENT AND IMPROVE THE FLAVOR.

OVEN

- ALLOWS EQUAL COOKING IN ALL PARTS OF THE FOOD.

- FOR MORE FLAVOR: BATHE WITH FAT-FREE LIQUIDS (CRUSHED NATURAL TOMATO, LEMON JUICE).

PAPILLOTE (ALTERNATIVE):

- WRAP THE FOOD IN VEGETABLE PAPER OR ALUMINUM FOIL.

- THE FOOD PRESERVES ITS PROPERTIES BETTER.

MICROWAVE

- CAN BE USED TO SOFTEN BEFORE COOKING.

- THE FOOD DOES NOT LOSE NUTRIENTS.

BOILED

- IMMERSE THE FOOD IN WATER.

- INCREASES DIGESTIBILITY.

- DOES NOT ADD CALORIES TO THE FOOD.

- AVOIDS LOSS OF NUTRIENTS (USE THE SOUP OR CREAM BROTH).

STEW

- INVOLVES USING OIL FIRST AND WATER/BROTH LATER.

- CONTROL THE AMOUNT OF OIL IN THE FRYING AND USE PLENTY OF BROTH.

- RESULTS IN VERY TASTY DISHES

STEAM

- ONLY STEAM IS USED TO COOK THE FOOD.

- CONTAINERS WITH 2 COMPARTMENTS (THE STEAM COMES OUT OF THE BOILING WATER AT THE BOTTOM).

- VERY HEALTHY AND EFFECTIVE METHOD: NO NUTRIENTS ARE LOST.

SESSION 4. TIPS FOR ADAPTING FOOD FOR LEISURE AND EXTRAORDINARY EVENTS

- MAINTAIN A REGULAR MEAL SCHEDULE; EAT 5 MEALS A DAY.

- DRINK 1.5 TO 2 LITERS OF WATER PER DAY.

- EAT 3 PIECES OF FRUIT PER DAY AND 2 PORTIONS OF VEGETABLES.

- EXERCISE AT LEAST 3 TIMES A WEEK AS APPROPRIATE FOR EACH PERSON.

- PLAN THE DIFFERENT MEALS.

- DINNER SHOULD BE OF LOWER CALORIC DENSITY (SALADS OR VEGETABLES WITH GRILLED MEAT OR FISH).

IN ADDITION, ON HOLIDAYS:

- DO NOT ARRIVE HUNGRY. HAVING A HIGH-PROTEIN SNACK (LOW-FAT YOGURT) BEFORE GOING TO A PARTY WILL HELP YOU AVOID OVEREATING.

- WHEN EATING A LARGE MEAL, FOLLOW A DIET BASED ON FRUITS (MAINLY CITRUS FRUITS: PINEAPPLE, KIWI, ORANGE JUICE, STRAWBERRIES,...), VEGETABLES FOR 2 OR 3 DAYS. PREFER WHITE MEAT (FISH OR CHICKEN BREAST FILLET) AND DRINK WATER THROUGHOUT THE DAY.

- CONTROL THE PORTIONS THAT ARE INGESTED, TRY EVERYTHING BUT NOT IN EXCESS.

- SELECT. AVOID THE FOODS THAT ARE RICHEST IN CALORIES, TRY THEM BUT CHOOSE THE HEALTHIEST FOODS.

FOOD GROUP	FREQUENCY	WEIGHT OF EACH RAW PORTION	HOMEMADE MEASURES ALREADY COOKED
Cereals, bread	2-3 s/d	50 g bread<br>60-80 g of pasta, rice	1/5 of a loaf of bread<br>1 normal single dish
Vegetables	> 2 s/w	150-200 g	1 plate of mixed salad, 1 plate of cooked vegetables
Fruits	> 3 s/d	120-200 g	1 medium piece of fruit
Olive oil	2 s/d	10 ml	1 tablespoon
Dairy products	2-4 s/d	200-250 ml milk<br>150 g yogurt, 40-60 g fresh cheese	1 cup of milk<br>1 yogurt<br>1 individual portion
Fish and seafood	3-4 s/wk	125-150 g	1 steak
Poultry and eggs	3 s/wk	100-125 g	1 steak, 1-2 eggs sem/wk
Legumes	2-4 s/wk	60-80 g	1 normal single dish

SESSION 6. FALSE BELIEFS AND MYTHS OF FOOD

WHOLEGRAIN FOODS ARE LESS FATTENING THAN THOSE THAT ARE NOT WHOLEGRAIN:

THE DIFFERENCE IN CALORIES BETWEEN WHOLEGRAIN FOODS AND NON-WHOLEMEAL FOODS IS MINIMAL, SO THEY PRACTICALLY "FATTEN" THE SAME.

THE DIFFERENCES ARE: BETTER DIGESTION, MORE FIBER, MORE SATIETY, MORE VITAMINS AND MINERALS

WHOLEMEAL BREADS ARE LESS FATTENING:

THEY HAVE PRACTICALLY THE SAME CALORIES, THOUGH IT IS HEALTHIER TO EAT WHOLE-GRAIN BREAD

POTATOES ARE FATTENING:

THE BAD THING IS THE OIL THAT USUALLY ACCOMPANIES THEM.

MARGARINE IS LESS FATTENING THAN BUTTER

BOTH HAVE PRACTICALLY THE SAME CALORIES

MARGARINE IS HEALTHIER BECAUSE ITS FAT IS OF VEGETABLE ORIGIN AND HAS NO CHOLESTEROL, AND MAY EVEN HELP REDUCE CHOLESTEROL

BROWN SUGAR IS BETTER THAN WHITE SUGAR

THE ONLY DIFFERENCE BETWEEN BOTH IS THAT WHITE GOES THROUGH A REFINING PROCESS, AND BROWN DOES NOT, HENCE THE COLOR DIFFERENCE.

BROWN SUGAR HAS A LITTLE MORE MINERALS.

BOTH ARE CARBOHYDRATES, AND PROVIDE 400 CALORIES PER 100 G.

SWEETENERS ARE CARCINOGENIC

ALL SWEETENERS ON THE MARKET ARE SAFE: THIS IS SCIENTIFICALLY PROVEN

THEY HAVE GREATER SWEETENING POWER THAN SUGAR AND FEWER CALORIES

THE USE OF SWEETENERS VERSUS SUGAR FOR WEIGHT CONTROL IS ADVISABLE

EATING FAST MAKES YOU FAT

THERE IS NO REASON WHY EATING FAST SHOULD AFFECT YOUR CALORIE INTAKE, AS LONG AS YOU EAT THE SAME AMOUNT.

IT TAKES YOUR MIND TO PROCESS THAT YOU ARE FULL, BUT IF YOU WAIT 20 MINUTES AFTER YOU HAVE EATEN, YOU WILL REALIZE THAT YOU DO NOT NEED TO EAT ANY MORE

WINE IS GOOD FOR THE HEART

SMALL AMOUNTS OF WINE (1/2 GLASS A DAY) CAN BE BENEFICIAL FOR THE BODY

IT IS AN ALCOHOLIC BEVERAGE: EXCESS IS HARMFUL

NON-ALCOHOLIC BEER IS NOT FATTENING

IT DOES PUT ON WEIGHT, BUT A LITTLE LESS, SINCE IT HAS FEWER CALORIES THAN NORMAL BEER. MOREOVER, EVEN IF IT SAYS "WITHOUT ALCOHOL" IT NORMALLY HAS AN ALCOHOLIC CONTENT (0.07%), AND THE SAME CALORIES AS MALT.

FRUIT AFTER MEALS IS FATTENING

FRUIT IS NOT FATTENING. IT CAN BE EATEN AT ANY TIME OF THE DAY.

IN ADDITION, THE VITAMIN C CONTAINED IN FRUIT ALLOWS, WHEN EATEN AS A DESSERT, A GREATER ABSORPTION OF THE IRON THAT COMES FROM THE MAIN DISH.

OLIVES ARE FORBIDDEN IN WEIGHT LOSS DIETS

OLIVES ARE NOT CONTRAINDICATED. THEY CAN BE USED AS EQUIVALENT. FOR EXAMPLE: INSTEAD OF USING A SPOONFUL OF OIL IN THE SALAD (WHICH IS EQUIVALENT TO 10 G AND 100 CALORIES) YOU CAN EAT 5 OR 6 NATURAL OLIVES (WITH PITS, NOT STUFFED).

SESSION 7. HANDLING AN EQUIVALENCY CHART

The equivalence tables have been classified according to the group to which each food belongs: rich in carbohydrates, rich in proteins or rich in fat. **FOODS RICH IN CARBOHYDRATES**

FOOD	APPROXIMATE MEASURE PER SERVING (EQUIVALENCE)	REPLACEABLE BY
**Rice**	Half a cup of coffee or half a scoop (about 50g)	Pasta, legumes, potatoes
**Pasta**	About 60g	Rice, legumes, potatoes
**Potatoes**	A middle potato (100-120g)	Rice, pasta, legumes
**Legumes**	2/3 cup of coffee or 2/3 scoop (about 60g)	Rice, paste, potato
**Cookies**	4-5 plain cookies, 2 digestive cookies (about 30g)	Cereals, bread
**Cereals**	3-4 tablespoons (30-40g)	Cookies, bread
**Bread**	1/6th of a quarter bar (about 40g)	Cookies, cereals

PROTEIN-RICH FOODS

FOOD	APPROXIMATE MEASURE PER SERVING (EQUIVALENCE)	REPLACEABLE BY
**Chicken or turkey breast**	2-3 fillets, depending on the thickness (about 150g)	Rabbit, beef
**Rabbit**	3-4 pieces, thigh or counter thigh (about 150g)	Chicken or turkey, beef
**Beef**	1-2 fillets, depending on thickness (about 120g)	Chicken or turkey, rabbit
**White fish**	3-4 fillets (about 120-200g)	Semi-fish, blue fish
**Semi-pigrassed fish**	1-2 fillets (about 100-180g)	White fish, blue fish
**Blue fish**	1-2 steaks (about 100-150g)	White fish, semi-fatty fish
**Egg**	1 egg or 2 whites and 1 yolk (50-100g)	Meat of any kind, fish of any kind
**Chicken or turkey sausage, cooked ham**	2-4 slices, 10-15 cubes	Tuna, bonito or tinned mackerel, surimi sticks, smoked salmon
**Tuna, bonito or mackerel (natural or drained)**	A can	Chicken or turkey sausage, ham, surimi sticks, smoked salmon
**Surimi sticks, smoked salmon**	2-3 sticks, 2-3 slices	Chicken or turkey sausage, ham, surimi sticks, smoked salmon

MOST RECOMMENDED FRUITS

FOOD	APPROXIMATE MEASURE PER SERVING (EQUIVALENCE)
**APRICOTS**	03 Units
**PLUMS**	03 Units
**CHERRIES**	125g
**KIWIS**	02 Units
**STRAWBERRIES**	125g
**MELON**	200g
**WATERMELON**	200g
**PINEAPPLE**	2 - 3 slices

NOTE: Compotes, fruit in syrup, dried fruit or juice are unhealthy

FATS

FOOD	APPROXIMATE MEASURE PER SERVING (EQUIVALENCE)	REPLACEABLE BY
**Olive oil**	One tablespoon (about 10g)	Nuts, olives, avocado
**Nuts**	A small handful (about 15-20g)	Olive oil, olives, avocado
**Olives (with bone)**	5-6 units	Olive oil, nuts, avocado
**Avocado**	¼ part (about 20-30g)	Olive oil, nuts, olives

SESSION 8. MACRONUTRIENTS AND MICRONUTRIENTS

MACRONUTRIENTS

- NUTRIENTS THAT SUPPLY MOST OF THE BODY'S METABOLIC ENERGY.
- THE MAIN ONES ARE: **LIPIDS, CARBOHYDRATES AND PROTEINS.**
- THEY DIFFER FROM MICRONUTRIENTS AS THEY ARE NECESSARY TO PRODUCE ENERGY

MICRONUTRIENTS

- REGULATORY FUNCTION: MINERALS AND VITAMINS (WATER-SOLUBLE AND FAT-SOLUBLE)
- ARE OBTAINED FROM FOOD: THE HUMAN BODY IS NOT CAPABLE OF PRODUCING THEM.
- MAIN SOURCES: FRUITS, VEGETABLES AND SALADS.
- ARE NEEDED IN MUCH SMALLER AMOUNTS THAN MACRONUTRIENTS.
- AN ADEQUATE EATING PLAN PROVIDES THE BODY WITH THE NECESSARY QUANTITIES.

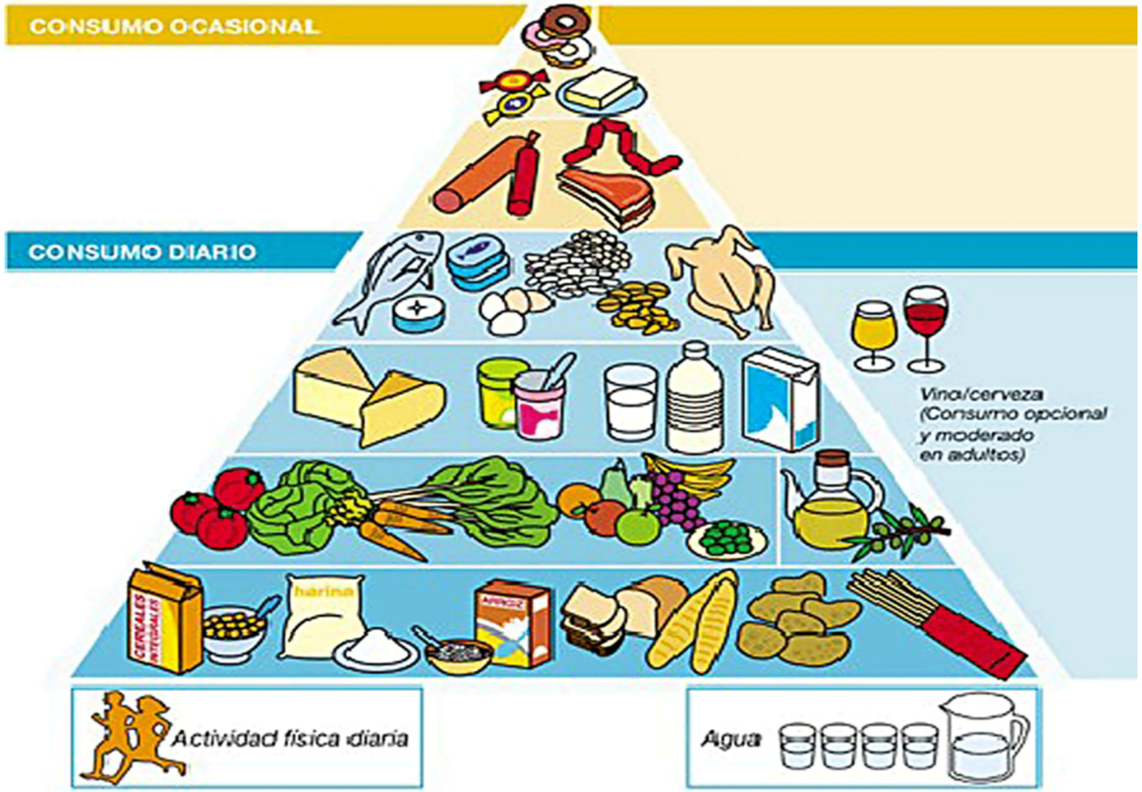

Pirámide SENC (2015)

SESSION 9. OBESITY AND OVERWEIGHT

BMI, formerly called the Quetelet index, is a measure for indicating nutritional status in adults. It is defined as a person's weight in kilograms divided by the square of the person's height in meters (kg/m2). For example, an adult that weighs 70 kg and whose height is 1.75 m will have a BMI of 22.9.

70 (kg)/1.752 (m2) = 22.9 BMI

For adults over 20 years old, BMI	**Nutritional status**
Below 18.5	Underweight
18.5–24.9	Normal weight
25.0–29.9	Pre-obesity
30.0–34.9	Obesity class I
35.0–39.9	Obesity class II
Above 40	Obesity class III

(WHO)

RISKS

- CHANGES IN RESPIRATORY FUNCTION (FATIGUE)

- INCREASES THE RISK OF CARDIOVASCULAR DISEASE (HIGH BLOOD PRESSURE, CORONARY ISCHEMIA...)

- INCREASED CHOLESTEROL.

- ORTHOPEDIC (MOTOR, BONE AND JOINT PROBLEMS)

- ADVERSE PSYCHOLOGICAL REACTIONS (LOW SELF-ESTEEM, DEPRESSION...), DEVELOPMENT OF RISK BEHAVIORS SUCH AS BULIMIA AND ANOREXIA NERVOSA, COMPULSIVE EATING OR SUICIDAL THOUGHTS

- HARMFUL EFFECTS ON HEART, VASCULAR AND METABOLIC DISEASES (HYPERLIPIDEMIA, DIABETES, GOUT...)

SESSION 10. MIRACLE DIETS

CHARACTERISTICS OF "MIRACLE DIETS":

- THEY OFFER QUICK WEIGHT LOSS WITH NO EXPERIENCE FOR THOSE WHO ARE ON THEM.

- LOW CALORIES THEY PROVIDE (VERY SEVERE CALORIC RESTRICTION), USUALLY ACCOMPANIED BY HARMFUL HABITS.

- MOST OF THESE DIETS ARE PRESCRIBED BY PEOPLE OUTSIDE THE FIELD OF NUTRITION.

- MIRACLE DIETS LOOK MORE FOR ECONOMIC BENEFIT THAN FOR HEALTHY AND BALANCED EATING.

DANGERS OF “MIRACLE DIETS”:

- FOOD MONOTONY THAT MAKES THEM UNSUSTAINABLE OVER TIME AND DANGEROUS FOR HEALTH.

- SITUATIONS OF VITAMIN AND MINERAL DEFICIENCIES.

- THE BODY REACTS BY COMPENSATING THE LACK OF ENERGY RECEIVED WITH AN INCREASE IN THE DESTRUCTION OF BODY PROTEINS, AS AN ALTERNATIVE SOURCE OF ENERGY.

SESSION 11. HEALTHY FATS

- **CLASSIFICATION OF FOOD FATS:**

❖ Saturated fat — NO HEALTHY

❖ Monosaturated Fat

❖ Polyunsaturated fat

HEALTHY

ARE CARDIO-HEALTHY

NUTRITIONAL OBJECTIVES:

TOTAL FAT: 30-35% (Total energy)

Saturated fat 7-8% (Total percentage)

Monounsaturated Fat 15-20% (Total percentage)

Polyunsaturated fat 5% (Total percentage)

Cholesterol < 300 mg/day

SESSION 12. READING NUTRITION LABELLING OF FOODS

"LABELLING MUST PROTECT THE CONSUMER AGAINST ABUSIVE BUSINESS PRACTICES"

- THE NUTRITION FACTS LABEL IS A SIMPLE TOOL THAT WE WILL FIND ON ALL BEVERAGES AND PACKAGED FOODS.

- UNDERSTANDING WHAT THE NUTRITION FACTS LABEL INCLUDES SHOULD HELP US DECIDE WHETHER TO BUY ONE PRODUCT OR ANOTHER.

- THE INDICATIONS MUST BE EASY TO UNDERSTAND, VISIBLE AND CLEARLY LEGIBLE, SO AS NOT TO MISLEAD THE BUYER.

- LABELS SHOULD NEVER STATE THAT A PRODUCT HAS CURATIVE PROPERTIES FOR A DISEASE.

- THE INFORMATION THAT IS PUT ON THE LABEL HAS TO BE TRUTHFUL AND RIGOROUS.

TERMS OF NUTRITION LABELLING

- FAT FREE: 0.5g OR LESS OF TOTAL FAT PER PORTION.
- LOW FAT: 3g OR LESS TOTAL FAT PER PORTION.
- REDUCED OR LESS FAT: 25% LESS TOTAL FAT, COMPARED TO THE ORIGINAL PRODUCT.
- TRANS FAT FREE: 0.5g OR LESS PER PORTION.
- LOW SATURATED FAT: 1g OR LESS PER PORTION.
- LOW CHOLESTEROL: 20MG OR LESS PER SERVING.
- SODIUM FREE: 5 MG OR LESS PER SERVING.
- LOW SODIUM: 140mg OR LESS PER SERVING.
- SUGAR FREE: 0.5g OR LESS SUGAR PER SERVING.
- LOW SUGAR: 25% LESS SUGAR, THAN THE ORIGINAL.
- NO SUGAR ADDED: NO SUGAR ADDED, ONLY CONTAINS THE NATURAL SUGAR OF THE FOOD. "SUGARLESS": INDICATED ON THE GUMS THAT DO NOT CONTAIN SUGAR.
- CALORIE-FREE: 5 CALORIES OR LESS PER SERVING.
- LOW CALORIE: 40 CALORIES OR LESS PER SERVING.
- LIGHT: ONE-THIRD OF THE CALORIES OR 50 PERCENT (%) LESS FAT COMPARED TO THE ORIGINAL PRODUCT.
- HIGH IN FIBER: 5g OR MORE PER SERVING
- GOOD SOURCE FIBER: 2.5g TO 4.9g PER PORTION.

SESSION 13. GUIDELINES FOR MAKING A HEALTHY PURCHASE

- MAKE A LIST BEFORE GOING SHOPPING
- AVOID PROCESSED FOODS
- REDUCE THE NUMBER OF VISITS TO THE SUPERMARKET
- DO NOT GO SHOPPING ON AN EMPTY STOMACH OR BEFORE MAIN MEALS
- DO NOT BUY THE PRODUCTS OFFERED AT THE CHECKOUT
- IGNORE PICTURES OF PRODUCT PACKAGING
- FORGET APPEARANCES, LOOK INSIDE
- READ THE LABELS OF THE PRODUCTS CAREFULLY
