# Supplementary material for: Improved Nutritional Knowledge in the Obese Adult Population Modifies Eating Habits and Serum and Anthropometric Markers
Source: Nutrients. 2020 Oct 30;12(11):3355. doi: 10.3390/nu12113355 (PMC7693073; doi:10.3390/nu12113355)
Supplement: Supplementary file 1 [file nutrients-12-03355-s001.zip › nutrients-975371-supplementary/Supplementary file 1.pdf]

nutrients

MDPI

Supplementary file 1

Session 1. Each participant is given the report with his/her nutritional assessment. Exercises to interpret the results using anonymous examples. Explanation of each parameter and its relation to lifestyles and repercussions upon health.

Session 2. General recommendations for an adequate food plan. Several anonymous eating plans were taken as a sample, and role-playing dynamics were set up for some participants for implementation, so that doubts could arise and be solved in the workshop.

Session 3. Different ways of cooking. Different recipes were presented with the different ways of healthy cooking of the different food groups. The participants were also encouraged to explain how they cook food, and a joint analysis was made of whether it is a healthy way of cooking or not.

Session 4. Adapting the food plan to vacations and extraordinary situations. Several situations were raised in which the participants would find it difficult to follow a healthy eating plan - with practical solutions being sought to overcome them. The participants were also encouraged to propose personal situations.

Session 5. Portions and equivalents. A batch was prepared with different products and kitchen tools, and the participants checked the approximate amount of each of them (spoon, teaspoon, glass, handful, etc.).

Session 6. Food myths. A series of myths were raised. It was asked whether they are considered to be true or false, and a reasoned explanation was given as to why they are or are not.

Session 7. Handling of food equivalency tables. The table was presented, and participants were encouraged to carry out exchange exercises with their usual meals.

Session 8. Macronutrients and micronutrients. We worked with the SENC food pyramid to teach participants which macronutrients and micronutrients are the most important components of the different food groups.

Session 9. Overweight and obesity. The concepts of overweight and obesity, and their repercussions upon health over the short, middle and long term were explained. Special attention focused on modifiable risk factors, in order to promote changes in lifestyle. The session was conducted using case studies prepared by the team.

Session 10. Miracle diets. The different current miracle diets and their effects upon health were described. The participants were organized into small groups of 5 people. A different diet was given per group, and the participants were encouraged to look for the reasons why they are not healthy. Reasons were then given as to why each of the diets is healthy or not.

Session 11. Heart-healthy fats. The different types of fats contained in food, and those that are heart-healthy, were explained. We worked again with the different food groups and with the processed foods that are usually available on sale.

Session 12. Nutrition labeling of foods. We worked with different types of real labels for commonly consumed foods.

Session 13. Healthy shopping. Guidelines for healthy shopping and correct reading of nutrition labeling were provided. A list of recommendations for making healthy purchases was given in the form of a card with spaces for adding new items, and later the group brainstormed ideas that, once analyzed by the professionals, enriched the initial list.

Session 14. Review of the contents of the workshops The participants were given a notebook with a summary of the contents worked upon in the workshops, and were proposed to make a tour that would start by

developing a healthy eating plan, a healthy purchase, and aA healthy cooking.. Moreover somestrategies for holiday periods or outside their usual routines

Session 15. Presentation of achievements and tips for maintaining healthy habits. Reports were given individually, and the achievements of the participants who volunteered during the program were analyzed and new commitments were made, with the aim of avoiding a return to the habits and behaviors that led them to excess body weight. To this end, the strategies learned were reviewed.
